# Predictive Biomarkers for Sustained Unresponsiveness Following Oral Immunotherapy

**DOI:** 10.1111/cea.70293

**Published:** 2026-03-31

**Authors:** Duan Ni, Ralph Nanan

**Affiliations:** ^1^ Sydney Medical School, Nepean, the University of Sydney Sydney New South Wales Australia; ^2^ Charles Perkins Centre, the University of Sydney Sydney New South Wales Australia; ^3^ Nepean Hospital, Nepean Blue Mountains Local Health District Sydney New South Wales Australia

1

**Summary**

- Baseline allergen‐specific IgE, particularly epitope‐specific IgE, is a robust predictor for sustained unresponsiveness following oral immunotherapy.
- Biologically informed, individualised OIT strategies may improve sustained unresponsiveness outcomes for food allergy.

To the Editor,

1

Oral immunotherapy (OIT) is currently the major curative approach for food allergy. Although most individuals receiving OIT develop *desensitisation* during active treatment, *sustained unresponsiveness (SU)* or *remission*, defined as the ability to tolerate food allergens after OIT completion, occurs only in a minority of recipients [1]. Therefore, to predict SU prior to OIT initiation is critical for stratifying patients and advancing precision medicine approaches in food allergy. Here, we present a narrative review with a structured literature search, interrogating recent advances in predicting SU after OIT and comparatively assessing proposed baseline predictors.

We exhaustively surveyed publications on PubMed with OIT clinical studies/trials related to predicting SU with baseline profiles. Literature search was performed using search terms “((allergy) OR (food allergy)) AND ((oral immunotherapy) OR (oral immune therapy) OR (OIT)) AND ((sustained unresponsiveness))” and “((allergy) OR (food allergy)) AND ((oral immunotherapy) OR (oral immune therapy) OR (OIT)) AND ((remission))”. Our search end date is 23rd February 2026. Aforementioned search terms retrieved 324 papers. After screening their titles and abstracts in detail, 22 papers with in‐depth analysis for SU/remission (referred to as SU hereafter for simplicity) in food allergy OIT were found for further analysis, encompassing 11 clinical studies across 5 common food allergens: peanut, egg, wheat, milk, and fish.

Among these reviewed studies, SU predictors can be broadly categorised as: immune parameters, gut microbiota profiles, and clinical measurements. Immune‐based markers were most commonly evaluated. Allergen‐specific IgE, particularly epitope‐specific IgE (e.g., Ara h 2 for peanut), generally exhibited stronger predictive performances, with significant differences reported between the *SU* and *no SU* groups, and comparatively higher areas under the curves (AUCs) in receiver operating characteristic (ROC) analyses. Other immune‐related parameters, including T cell activation profiles and basophil responsiveness to allergens, showed weaker predictive performance. For example, baseline basophil activation and sensitivity assay results could not predict SU outcomes in [2].

Gut microbiota‐based predictors exhibited modest predictive capacity. Özçam et al. reported that microbiota phylogenetic diversity and metabolomic features differentiated *SU* vs. *no SU* groups with a lower ROC AUC (0.712) [3]. Clinical measurements, including age, skin prick test wheal size, and allergen cumulative reactive dose, also showed associations with SU, but their predictive performance appeared less potent compared with allergen‐specific IgE measures, which seemed to be the best predictors across all reviewed studies.

Importantly, applications of advanced mathematical methods like machine learning (ML), where complex data are available, can substantially improve predictive power. In the POISED trial, an ML‐based model incorporating allergen‐ and epitope‐specific IgE profiles yielded an ROC AUC of 0.99 [4].

Statistical measurements to evaluate predictive performances vary in the aforementioned publications. To enable cross‐sectional comparisons, we re‐analysed 3 studies where source data were publicly available using ROC analysis with the *pROC* (v1.18.0) package based on each parameter and compared their ROC AUCs. With this approach, we confirmed that allergen‐ and epitope‐specific IgE consistently exhibited the best predictive performances, reaching ROC AUCs up to 0.92, in contrast to all other parameters with ROC AUCs below 0.75 (Table 1).

**TABLE 1:** Receiver operating characteristic (ROC) analyses for 3 studies with publicly available source data and the *Oral Peanut Immunotherapy with Butyrate Adjuvant* (OPIA, ACTRN12617000914369) clinical trial.

ROC analyses for 3 studies with publicly available source data							
Clinical trial ID	Allergen	Study design	Baseline measurement	Statistical output	# participants	Methods	References
IRCT20190612043872N1	Wheat	14–17 m OIT, 3–4 m avoidance	Wheat sIgE	AUC = 0.9231 (0.799, 1) (95% CI)	13 SU, 6 no SU	ImmunoCAP	[8]
NCT01867671	Peanut	IMPACT trial 134w OIT, 26w avoidance	Gut microbiota phylogenetic diversity	AUC = 0.7419 (0.5586, 0.9253)	16 SU, 31 no SU	Gut microbiota metagenomic analysis	[3]
			Peanut sIgE	AUC = 0.8558 (0.7405, 0.9712)		ImmunoCAP	
			Ara h 2 sIgE	AUC = 0.8344 (0.7069, 0.9620)			
NCT02103270	Peanut	POISED trial PN‐0: 2y OIT, 13w avoidance timepoint	Peanut‐stimulated %CD57+ of EM CD8+ T	AUC = 0.6914 (0.5346, 0.8482)	20 SU, 29 no SU	CyTOF	[9]
			Peanut‐stimulated %CD57+ of EMRA CD8+ T	AUC = 0.5957 (0.4345, 0.7569)	20 SU, 29 no SU		
			Peanut‐stimulated %naive CD8+ T of total live cell	AUC = 0.7302 (0.5885, 0.8718)	20 SU, 29 no SU		
			Unstimulated %CD57+ of EM CD8+ T	AUC = 0.6654 (0.5034, 0.8274)	20 SU, 26 no SU		
			Unstimulated %CD57+ of EMRA CD8+ T	AUC = 0.5913 (0.4234, 0.7593)	20 SU, 26 no SU		
			Unstimulated %naive CD8+ T of total live cell	AUC = 0.7240 (0.5770, 0.8710)	20 SU, 26 no SU		
			Peanut‐stimulated %CD86+ of memory B	AUC = 0.6784 (0.5159, 0.8410)	20 SU, 29 no SU		
			Unstimulated %CD86+ of classical monocyte	AUC = 0.6837 (0.5270, 0.8403)	20 SU, 26 no SU		
			Unstimulated %CD86+ of mDC2	AUC = 0.6010 (0.4340, 0.7679)	20 SU, 26 no SU		
			Peanut‐stimulated %CD57+ of CD56dimCD16+ NK cell	AUC = 0.6388 (0.4719, 0.8057)	20 SU, 29 no SU		
			Unstimulated %CD57+ of CD56dimCD16+ NK cell	AUC = 0.6481 (0.4795, 0.8167)	20 SU, 26 no SU		
			Peanut sIgE	AUC = 0.7986 (0.6451, 0.9552)	20 SU, 29 no SU	ImmunoCAP	
			Ara h 2 sIgE	AUC = 0.8287 (0.6789, 0.9785)	20 SU, 29 no SU		
			Ara h 2 sIgG4	AUC = 0.5567 (0.3613, 0.7521)	20 SU, 29 no SU		
			Ara h 2 sIgE/Ara h 2 sIgG4	AUC = 0.9190 (0.8141, 1.000)	20 SU, 29 no SU		
ROC analyses for the in‐house *Oral Peanut Immunotherapy with Butyrate Adjuvant* (OPIA, ACTRN12617000914369) clinical trial							
ACTRN12617000914369	Peanut	OPIA trial 1y OIT supplemented with HAMSB/LAMS, 6w avoidance (HAMSB, LAMS arms combined for analysis)	Peanut eliciting dose	AUC = 0.7315 (0.5940, 0.8689)	20 SU, 27 no SU	Clinical measurements	[5, 6]
			Peanut cumulative reactive dose	AUC = 0.7370 (0.5992, 0.8749)			
			Peanut‐stimulated %IFNγ+ of CD4 + CD154+ pr effector T cell	AUC = 0.6481 (0.4913, 0.8049)		Spectral flow cytometry	
			Peanut‐stimulated %IL‐4+ of CD4 + CD154+ pr effector T cell	AUC = 0.6722 (0.5088, 0.8356)			
			Peanut‐stimulated %IL‐5+ of CD4 + CD154+ pr effector T cell	AUC = 0.6806 (0.5212, 0.8399)			
			Peanut‐stimulated %IL‐10+ of CD4 + CD154+ pr effector T cell	AUC = 0.6046 (0.4362, 0.7730)			
			Peanut‐stimulated %IL‐13+ of CD4 + CD154+ pr effector T cell	AUC = 0.7000 (0.5449, 0.8551)			

Abbreviations: AUC: area under the curve; CyTOF: cytometry by time of flight; EM: effector memory; EMRA: effector memory re‐expressing CD45RA; HAMSB: butyrylated high amylose maize starch; LAMS: low amylose maize starch; mDC2: type 2 myeloid dendritic cell; NK cell: natural killer cell; pr: peanut reactive; sIgE: specific IgE; sIgG4: specific IgG4.

We also carried out similar comparisons using our own *Oral Peanut Immunotherapy with butyrate Adjuvant* (OPIA, ACTRN12617000914369) clinical trial cohort [5, 6]. The OPIA study was a randomised double‐blinded, placebo‐controlled trial led by our team as previously described [5, 6]. The OPIA trial contained 3 arms: no OIT, 12‐month OIT with butyrylated high amylose maize starch (HAMSB) as an adjuvant, and 12‐month OIT with placebo low amylose maize starch (LAMS). For the current analysis, we focused on the 2 OIT treatment arms (HAMSB and LAMS) and combined them for comparisons. Analyses on the OPIA trial cohort found that clinical measurements like peanut eliciting and cumulative reactive doses seemed to outperform immune parameters like peanut‐reactive T cell functional profiles in predicting SU (Table 1).

Collectively, we here present the most comprehensive synthesis to date of state‐of‐the‐art advances in predicting SU following OIT. Overall, allergen‐ and epitope‐specific IgE consistently emerged as the strongest predictor of SU, outperforming other immune‐ and microbiota‐related parameters. Clinical measurements, including skin prick testing and food challenges, also exhibited robust predictive power. Importantly, these findings were confirmed through comprehensive reanalyses of 3 publicly available study datasets and in our OPIA clinical trial, although only a subset of representative OPIA data is included here, with additional analyses for more baseline variables (e.g., age and baseline demographics) to be reported separately.

As a narrative review, this study is inherently susceptible to bias related to study selection and the nature of the analytical approach. More comprehensive systematic reviews and meta‐analyses are therefore warranted to address these limitations.

Accurate prediction of SU is of substantial clinical and research relevance. However, feasibility, cost and scalability are also of critical consideration for implementation in routine practice and for research. In this regard, allergen‐specific IgE measurement seems to be superior, because it exhibits robust predictive power and is cost‐effective and already available. Importantly, the predictive performances of allergen‐specific IgE may be further strengthened when integrated with complementary clinical variables, as suggested by prior studies [4]. In addition, treatment‐related factors during OIT, such as adherence (e.g., missed doses) and dosing‐related adverse outcomes, are likely to influence OIT outcomes as well [7]. Future predictive frameworks may therefore benefit from incorporating both baseline immune markers and longitudinal treatment variables.

Currently, most OIT protocols apply limited patient stratifications to fine‐tune their dosing strategies and/or treatment durations. Our findings suggest that baseline measurements like allergen‐ and epitope‐specific IgE profiles could facilitate more tailored approaches to move the field towards precision‐guided OIT, enabling individualised risk stratification and enhancing SU outcome prediction.

## Author Contributions

Concept and design and drafting of the manuscript: Duan Ni and Ralph Nanan. Acquisition, analysis and interpretation of data: Duan Ni and Ralph Nanan. Both authors read and approved the final manuscript.

## Funding

This work was supported by the National Health and Medical Research Council (NHMRC 1104134) and Norman Ernest Bequest Fund. The OPIA trial is funded by a National Health and Medical Research Council Australia Project Grant (NHMRC 1104134). D.N. was supported by the Norman Ernest Bequest Fund.

## Ethics Statement

The OPIA trial was registered on 22 June 2017 in the Australian New Zealand Clinical Trials Registry as ACTRN12617000914369. The study was approved by the Human Research Ethics Committee of the Sydney Children's Hospital Network (HREC/16/SCHN/372). Written informed consent was obtained from parents/guardians, and assent was obtained.

## Conflicts of Interest

The authors declare no conflicts of interest.

## Data Availability

The data that support the findings of this study are openly available in GitHub at https://github.com/Nidane/SustainedResponsivenessPredictor.Online Repository data include a list of publications reviewed in current study and a table summarizing the state‐of‐the‐art advances in predicting SU outcome from OIT with baseline measurements based on 22 papers across 11 clinical trials/studies.
